# Primed for Reading

**DOI:** 10.1371/journal.pbio.1000424

**Published:** 2010-09-07

**Authors:** Robert Boyd

**Affiliations:** Department of Anthropology, University of California Los Angeles, Los Angeles, California, United States of America

**Figure pbio-1000424-g001:**
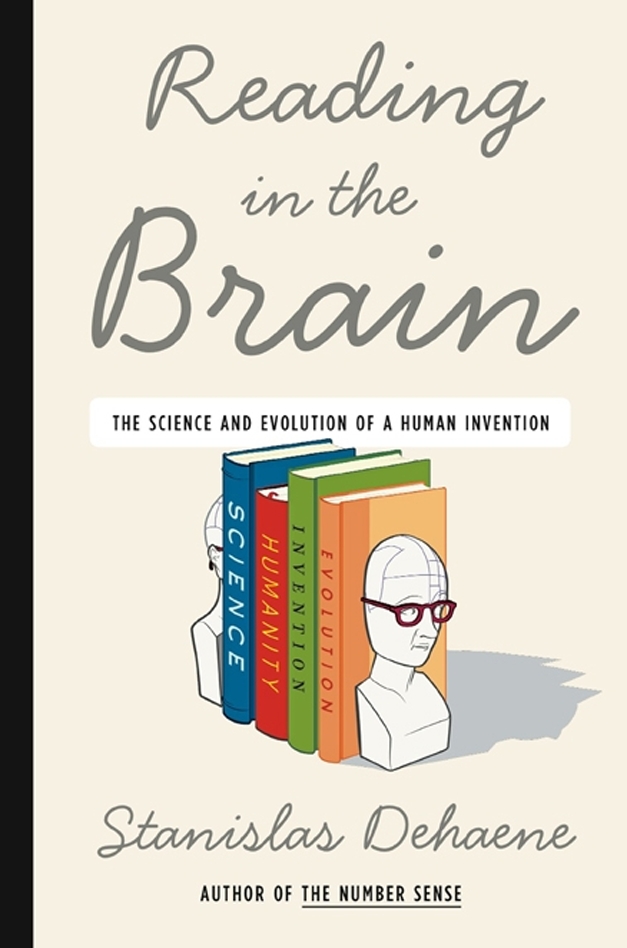
Dehaene S (2009) Reading in the Brain. New York: Penguin, Viking Adult. 400p. ISBN 978-0-670-02110-9. US$27.95.

[Fig pbio-1000424-g001]Reading is an amazing skill. As you read this review, meaning flows from the page (or for many readers, the screen) into your brain. This happens automatically—you can't choose not to understand the written word any more than the spoken one. It's also highly efficient. Most people can process text two or three times faster than speech. Of course, humans have many amazing skills. We also identify objects, decode speech, and understand complex social situations automatically and efficiently. However, the machinery in the brain that gives us these abilities, and many more, was plausibly constructed by natural selection, and, if so, they are adaptations just like our peculiar pelvis and the thick enamel on our molars. Reading arose a few thousand years ago, and this means that machinery in the brain that allows us to read did not evolve for that purpose. Instead, a series of scribes, priests, and printers working over a few thousand years gradually devised the writing systems that give rise to this amazing skill. In this fascinating book, Stanislaus Dehaene details how cognitive abilities evolved for other purposes were co-opted for reading, how these abilities are instantiated in the brain, and how they constrain the cultural evolution of writing systems.

Dehaene does an excellent job explaining how reading works at both the neurobiological and cognitive levels. He takes the reader seriously, laying out diverse kinds of evidence that bear on the problem. For example, when you read, visual information is shunted to a small region in the left hemisphere of your brain, the brain's “letter box” where the text is decoded. The earliest evidence for this came from the autopsy of a 19th century French stroke victim who lost the ability to read, even though he could still recognize numerals. More recently, PET and fMRI imaging studies have pinned down the location. Amazingly, these results show that it doesn't matter whether you read Italian or Chinese, the same part of the brain is involved.

Electroencephalograms gave us a better temporal resolution, and single neuron recordings of patients undergoing surgery confirm that only some neurons respond to text while others respond to faces, tools, and a myriad of other things. Information from the letter box then flows to brain regions that deal with speech and meaning, leading to a two-path system that allows us to rapidly recognize the meaning of familiar words while simultaneously sounding out unfamiliar ones. Dehaene tells the story experiment by experiment—a good narrative strategy because he excels at giving the reader an intuition for how the experiments work. For example, he likens diffusion fMRI to detecting roadways by looking at blurred nighttime photographs of the tail lights of the cars driving on them.

*Reading in the Brain* explains, with exceptional clarity, how machinery evolved for other purposes allows us to read and why this machinery constrains the form of writing systems. Organisms have to be able to recognize a wide variety of novel objects, and, accordingly, the letter box is in the region of the brain where object recognition takes place. There, different populations of neurons respond to an alphabet of elementary shapes, and correlations among these populations are used to identify objects. It turns out that the symbols used in all of the world's writing systems are closely related to these elementary shapes. Objects must be recognized at different distances and in different positions, so our object recognition system is insensitive to size and location. The same is true of all the world's writing systems. Once letters are recognized, they must be grouped into words. The first step in this process of comprehending alphabetic writing systems is the presence of neurons that respond to ordered pairs of letters, or “bigrams.” Correlations among bigrams are used to identify words (explaining why the acronym for French Connection UK is so arresting). At each step, the nature of the way that brain systems evolved for other tasks constrains the nature of writing systems.

This fact leads Dehaene to take a strongly nativist stance. Contrary to “social scientists,” he argues, the fact that our brains constrain the kinds of writing systems we can learn falsifies the belief that culture is completely unconstrained by biology. Instead, he argues, the structure of culture is determined by the innate machinery of the human mind—only cultural items that fit with this machinery can spread and persist. Undoubtedly, there are social scientists who believe that humans are a blank slate, but his beautifully detailed account of how reading works is not necessary to refute them. Reading is a tool, and tools are always constrained by the properties of the creature that uses them. Cultural evolution will not give rise to axes with 30cm diameter handles because of the structure of human hands or musical instruments that only produce sounds with frequencies above 20,000 Hz, because of the structure of human hearing. It is of great interest to know how brain systems constrain writing systems, but the fact that they do should not be the least bit surprising.

Discussions of culture and biology tend to have a Manichean flavor. Either culture is the evoked product of evolved cognitive modules, or it is something *sui generis*, uninfluenced by the brain. This book wonderfully demonstrates that culture is deeply rooted in biology of the human brain, and, at the same time, culture gives rise to novel, highly adaptive behaviors whose crucial functional details do not arise from the evolved properties of the brain, but rather are created by gradual cultural accumulation. In most past human environments, the brain's letter box allowed people to recognize people and objects, to distinguish friends from foes and axes from hammers. However, in environments in which people experience written texts, and in which there are institutions that help people learn how to decode these texts, this part of the brain develops into a reading machine that recognizes letters, groups them into bigrams, and ultimately into words. This information is then linked to other brain structures and the result is the capacity to rapidly and automatically read the written word, a powerful adaptive ability that transforms human minds and human societies. That brain machinery evolved for the purpose of object recognition is necessary for reading, but to understand why we read, it is also important to understand how cultural evolution shapes human environments to create completely novel and highly adaptive behaviors.

